# Aldol elaboration of 4,5,6,7-tetrahydroisoxazolo[4,3-*c*]pyridin-4-ones, masked precursors to acylpyridones

**DOI:** 10.3762/bjoc.8.33

**Published:** 2012-02-27

**Authors:** Raymond C F Jones, Abdul K Choudhury, James N Iley, Mark E Light, Georgia Loizou, Terence A Pillainayagam

**Affiliations:** 1 Department of Chemistry, Loughborough University, Loughborough, Leics. LE11 3TU, United Kingdom; 2 Department of Chemistry & Analytical Sciences, The Open University, Walton Hall, Milton Keynes MK7 6AA, United Kingdom; 3 EPSRC National Crystallography Service, School of Chemistry, University of Southampton, Highfield, Southampton, SO17 1BJ, United Kingdom

**Keywords:** aldol reaction, fused-ring systems, heterocycles, isoxazole, metalation, pyridone

## Abstract

A core 4,5,6,7-tetrahydroisoxazolo[4,3-*c*]pyridine-4-one scaffold is elaborated at C-3(Me) by base-mediated aldol condensation to give new 3-alkenyl-4,5,6,7-tetrahydroisoxazolo[4,3-*c*]pyridine-4-ones, which are masked forms related to the acylpyridone natural products.

## Introduction

**Figure 1:**
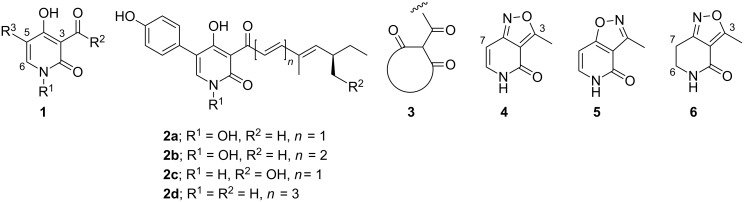
The 3-acyl-4-hydroxypyridin-2-one motif, example natural products and the isoxazolopyridone scaffolds.

The 3-acyl-4-hydroxypyridin-2-one moiety **1** (Figure 1) is the common structural unit of a family of natural products with a range of interesting biological activities [1]. Examples are the pigments tenellin (**2a**) and bassianin (**2b**) from insect pathogenic fungus *Beauveria bassiana* [2–3], pyridovericin (**2c**) [4] (a tyrosine kinase inhibitor) and the elfamycin antibiotics [5]. Interest has also been stimulated in these metabolites by the use of the entomopathogenic fungi such as *Cordyceps sp.*, many of which contain pyridone metabolites, in traditional Chinese medicine to strengthen the immune system and improve cognitive function. Farinosone A (**2d**) from *Paecilomyces farinosus*, for example, induces and enhances neurite outgrowth in the PC-12 cell line, although it is not clear whether the pyridones in general display neuritogenic properties [6]. The biosynthesis of tenellin and bassianin in *Beauveria bassiana* has recently been studied in detail by using genetic techniques, and been shown to involve conversion from an acyltetramic acid by oxidative ring expansion [7–8].

During a programme of synthesis towards metabolites containing the enolised heterocyclic tricarbonyl motif **3** [9–16], we have reported nitrile oxide dipolar-cycloaddition strategies to access the isoxazolo[4,3-*c*]pyridin-4-one **4** as 2^nd^-generation masked nonpolar scaffolds for the 3-acyl-4-hydroxypyridin-2-one nucleus [12]; our 1^st^-generation approach had employed the [4,5-*c*] isomer **5** [13–15]. We have recently reported on elaboration of isoxazolopyridone **4** at C-3(Me) and C-7 towards natural products and analogues [16]. An intermediate en route to scaffold **4** is the 4,5,6,7-tetrahydroisoxazolo[4,3-*c*]pyridin-4-one **6** [12,16], and the availability of this compound, combined with an interest in the biological potential of the acylpyridones and their dihydro derivatives (which are also ring homologues of bioactive 3-acyltetramic acid metabolites), led us to explore elaboration of this 6,7-dihydro-scaffold at C-3(Me) to produce masked forms of the acyldihydropyridones. We report here on these findings.

## Results and Discussion

**Scheme 1:**
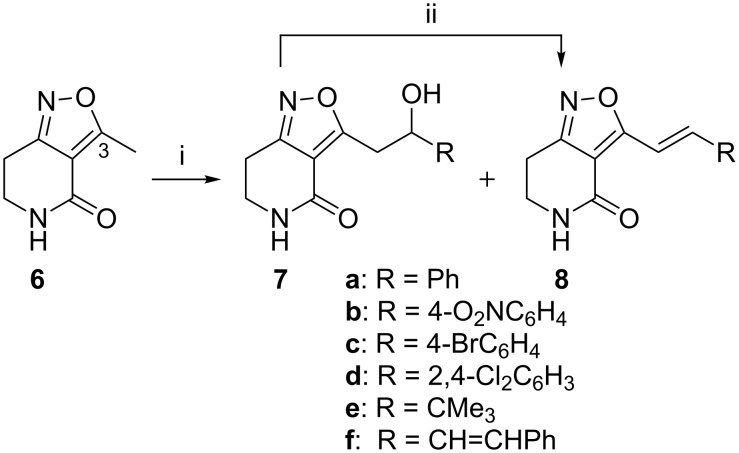
Aldol reactions of tetrahydroisoxazolopyridone **6**. Reagents: (i) LDA–TMEDA, RCHO, THF, −20 °C; (ii) toluene, reflux, PTSA.

**Figure 2:**
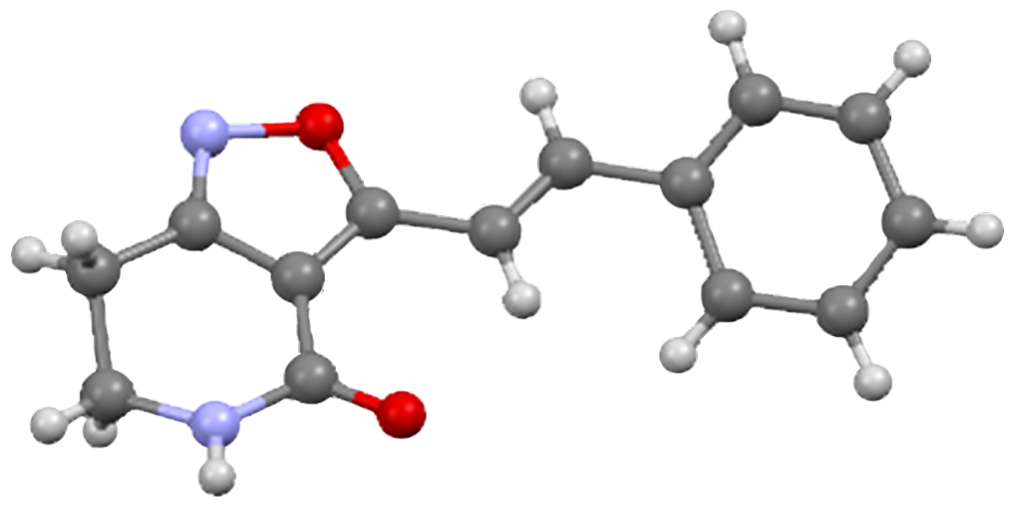
X-ray crystal structure of 3-(2-phenylethenyl)-4,5,6,7-tetrahydroisoxazolo[4,3-*c*]pyridin-4-one (**8a**) [19].

Our approach to the elaboration of the tetrahydroisoxazolopyridone **6** was to use the direct deprotonation strategy employed with the corresponding dehydro derivative **4** [16]. It is well-precedented that 3,5-disubstituted isoxazoles undergo lateral deprotonation–metalation preferentially at the C-5 substituent [17–18] (isoxazole numbering), which corresponds to the C-3 substituent in isoxazolopyridine **6**. We planned to undertake aldol-type reactions to lead to 3-alkenylisoxazolopyridones as masked forms of the corresponding acylpyridones, in which 3-alkenoyl substituents feature prominently, see Figure 1. Thus the 3-methyl compound **6** was treated with LDA–TMEDA (2.1 mol equiv of each; THF, −20 °C ice–salt bath) before addition of benzaldehyde (3.5 mol equiv; THF, −20 °C). After 1 hour at −20 °C the mixture was allowed to warm to 20 °C and stirred for 4 days. After this time, we were pleased to be able to isolate the condensation product, 3-(2-phenylethenyl)tetrahydroisoxazolopyridone **8a** (53%) rather than the presumed intermediate 3-(2-hydroxy-2-phenylethyl) compound **7a** (Scheme 1), along with unchanged starting material **6** (17%). ^1^H NMR studies indicated the newly formed alkene bond to have the expected *E*-configuration, with ^3^*J*_CH=CH_ = 16.5 Hz. The structure of the 3-alkenyl compound **8a** was confirmed by an X-ray crystallographic analysis (Figure 2, Supporting Information File 1 for crystal data) [19].

By using the same protocol, the reaction was repeated with 4-nitrobenzaldehyde to afford **8b** (52%). 4-Bromobenzaldehyde gave condensation product **8c** (15%) with hydroxy-adduct **7c** as the major product (35%); 2,4-dichlorobenzaldehyde similarly afforded **8d** (22%) and **7d** (51%), and the nonenolisable 2,2-dimethylpropanal led to **8e** in low yield (4%) along with adduct **7e** (55%). 3-Phenylpropenal gave condensation product **8f** but in a poor yield (12%) along with recovered starting material **6** (14%). These results are summarized in Table 1. In all cases the new alkene bonds have *E*-configuration (^3^*J*_CH=CH_ = 16.5–16.8 Hz). The less electrophilic 2,4-dimethoxy- and 4-dimethylaminobenzaldehydes did not react with the C-3(Me) anion. The hydroxy adducts **7c–e** could be made to undergo elimination under Dean–Stark conditions of water removal (toluene, reflux, PTSA in 2 portions; for quantities see Supporting Information File 1) to deliver more of the condensation products **8c–e** (41, 38, 44%, respectively, Table 1, see Supporting Information File 1 for full experimental and spectroscopic data).

**Table 1:** Preparation of 3-alkenylisoxazolopyridones **8**, hydroxy adducts **7**, and dehydration of adducts **7**.

R	Aldol reaction products **7** and **8**^a^		Dehydration of adducts **7**^b^
	3-Alkenyl compounds **8** (yield %)	Hydroxy adducts **7** (yield %)	3-Alkenyl compounds **8** (yield %)

Ph	**8a** (53%)	**7a** (–)	**–**
4-O_2_NC_6_H_4_	**8b** (52%)	**7b** (–)	**–**
4-BrC_6_H_4_	**8c** (15%)	**7c** (35%)	**8c** (41%)
2,4-Cl_2_C_6_H_3_	**8d** (22%)	**7d** (51%)	**8d** (38%)
CMe_3_	**8e** (4%)	**7e** (55%)	**8e** (44%)
CH=CHPh	**8f** (12%)	**7f** (–)	**–**

^a^Reagents: LDA–TMEDA, RCHO, THF, −20 °C; ^b^Reagents: toluene, reflux, PTSA.

As an alternative to the LDA protocol described above, the tetrahydroisoxazolopyridone **6** was deprotonated, by using BuLi (2.3 mol equiv; THF–hexanes, −78 °C), followed by addition of benzaldehyde (1.5 mol equiv) to afford the hydroxy adduct **7a** (70%). Dehydration was accomplished under Dean–Stark conditions (toluene, reflux, PTSA, 1.3 mol equiv) to give the 3-alkenyl compound **8a** (63%). This revised protocol has not yet been extended to the reaction of pyridone **6** with other aldehydes, but has been routinely employed for elaboration of the dehydro version **4** [16].

**Figure 3:**
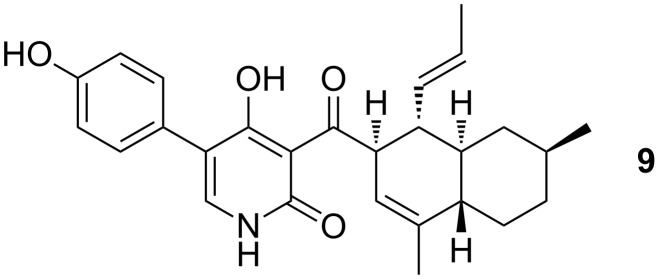
Ilicicolin H as an example of a 3-decalinoyl-4-hydroxypyridin-2-one metabolite.

**Scheme 2:**
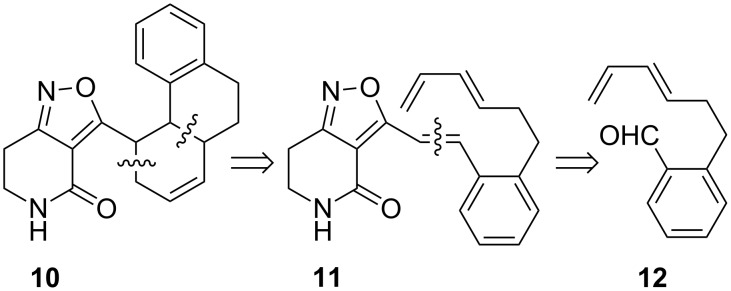
Retrosynthetic analysis of a model 3-decalinyl-4,5,6,7-tetrahydroisoxazolopyridone **10**.

The acylpyridone antifungal natural product ilicicolin H (**9**) (Figure 3) [20–21] along with fischerin, apiosporamide and YM-215343, displays a 3-decalinoyl-4-hydroxypyridin-2-one skeleton wherein the decalin unit may arise biosynthetically from a Diels–Alder reaction within the polyketide-derived side chain [1,22]. As a further demonstration of the aldol methodology, we proposed to construct the precursor triene **11**, which would facilitate a biomimetic cycloaddition approach to a benzo-fused decalin-substituted isoxazolopyridone, such as **10** (Scheme 2).

**Scheme 3:**
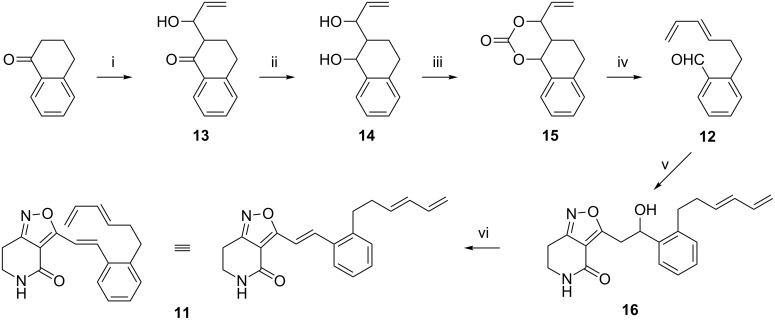
Synthesis of triene **11**. Reagents: (i), LDA, propenal, THF, −78 °C; (ii), LiAlH_4_, THF, 20 °C (75%); (iii), MeOCOCl, Et_3_N, CH_2_Cl_2_ (74%); (iv), Pd_2_(dba)_3_·CHCl_3_, MeCN, 20 °C (65%); (v), BuLi, isoxazolopyridone **6**, THF–hexanes, −78 °C (53%); (vi), toluene reflux, PTSA (78%).

The aldehyde **12** required for the 3-alkenyl side chain of triene **11** was prepared in four steps by the route shown in Scheme 3, involving palladium-catalysed decarboxylative ring opening of cyclic carbonates [23]. Thus the enolate of α-tetralone was added to propenal in an aldol addition (LDA, THF, −78 °C; 86%). The second step involved the reduction of the intermediate β-hydroxyketone **13**, (LiAlH_4_, THF) to give the 1,3-diol **14** in 75% yield. This was followed by synthesis of the cyclic carbonate **15** by treating the 1,3-diol **14** with methyl chloroformate (Et_3_N, CH_2_Cl_2_; 74%). In the final step, carbonate **15** was treated with palladium dibenzylideneacetone in dry MeCN at room temperature to afford the desired aldehyde **12** in 65% yield. Reaction of this aldehyde (10 mol equiv) with tetrahydroisoxazolopyridone **6** by using BuLi as a base (4.8 mol equiv; THF–hexanes, −78 °C) led to the aldol-type adduct **16** in 53% yield with some of the dehydrated condensation product also isolated as a minor product. This dehydration product, 3-alkenylisoxazolopyridone **11**, was prepared from the hydroxy adduct **16** by heating under a Dean–Stark water separator (toluene, reflux, PTSA, 1.5 mol equiv; 78%).

## Conclusion

We have thus developed suitable protocols for C-3 elaboration of the 3-methyl-4,5,6,7-tetrahydroisoxazolo[4,3-*c*]pyridin-4-one (**6**) to give 3-alkenyl derivatives **8** and **11**. These are masked forms of the corresponding 3-acyl-4-hydroxy-1,2,5,6-tetrahydropyridin-2-ones, which are of interest as dihydro relatives of the bioactive acylpyridone natural products and homologues of acyltetramic acids. Reductive methods to reveal the cyclic tricarbonyl moiety in such compounds have been reported by us previously [9–16].

## Supporting Information

Supporting information features full experimental and spectroscopic details for alkenylisoxazolopyridones **8a**–**f**, hydroxy adducts **7c**–**e**, compounds **11**–**16** and crystallographic data for **8a** (Figure 2).

File 1 Experimental and spectroscopic details.
