# Supplementary material for: Aldol elaboration of 4,5,6,7-tetrahydroisoxazolo[4,3-c]pyridin-4-ones, masked precursors to acylpyridones
Source: Beilstein J Org Chem. 2012 Feb 27;8:308–12. doi: 10.3762/bjoc.8.33 (PMC3302094; doi:10.3762/bjoc.8.33)
Supplement: File 1 — Experimental and spectroscopic details. [file Beilstein_J_Org_Chem-08-308-s001.pdf]

Supporting Information

for

Aldol elaboration of 4,5,6,7-tetrahydroisoxazolo[4,3-*c*]pyridin-4-ones, masked precursors to acylpyridones

Raymond C. F. Jones*[1], Abdul K. Choudhury[1], James N. Iley[2], Mark E. Light[3], Georgia Loizou[2] and Terence A. Pillainayagam[1]

Address: [1]Department of Chemistry, Loughborough University, Loughborough, Leics. LE11 3TU, United Kingdom, [2]Department of Chemistry & Analytical Sciences, The Open University, Walton Hall, Milton Keynes MK7 6AA, United Kingdom and [3]EPSRC National Crystallography Service, School of Chemistry, University of Southampton, Highfield, Southampton, SO17 1BJ, United Kingdom

Email: Raymond C. F. Jones - r.c.f.jones@lboro.ac.uk

* Corresponding author

Experimental and spectroscopic details

Experimental

General: Melting points were determined by using a Kofler hot-stage apparatus or an Electrothermal-IA 9100 apparatus, and are uncorrected. Infrared spectra were recorded on Perkin-Elmer 1710 FT or Paragon 1000 spectrometers. ^{1}H and ^{13}C NMR spectra were recorded on a Jeol JNM-LA300 spectrometer at 300 and 75 MHz, respectively, at the Open University, or a Bruker DPX 400 MHz spectrometer at 400 and 100 MHz, respectively, at Loughborough University. NMR spectra were determined in $CDCl_3$ solution (except where indicated) and chemical shifts are quoted in parts per million (ppm) from Me_4Si as internal standard. ^{1}H coupling constants *J* are quoted in Hz with multiplicities: s (singlet), d (doublet), t (triplet), q (quartet) and m (multiplet). The prefix br (broad) is used where applicable. Mass spectra were recorded either on a Joel SX-102 spectrometer (FAB, EI), or the Thermo Exactive (Orbitrap) accurate-mass spectrometer (ESI), fitted with a Triversa Advion Nanomate sample delivery system by using nano-ESI of methanol or methanol (1% w/w acetic acid) at Loughborough University, or were carried out by the EPSRC National Mass Spectrometry Service Centre (Swansea). Microanalytical data were measured on an Exeter Analytical CE440 Elemental Analyser at Loughborough University, or provided by Medac Ltd analytical and chemical consultancy services. Column chromatography was carried out at medium pressure by using Merck Kieselgel 60 (Art. 9385). Thin-layer chromatography was carried out on silica plates (Kieselgel 60, F254, Merck Art. 554). PE refers to the light petroleum fraction having bp 40–60 °C. Solvents were distilled before use: PE and EtOAc from $CaCl_2$, CH_2Cl_2 from CaH_2, THF freshly distilled from sodium and benzophenone under an atmosphere of nitrogen, MeOH freshly distilled from $Mg(OMe)_2$.

3-(2-Phenylethenyl)-4,5,6,7-tetrahydroisoxazolo[4,3-*c*]pyridin-4-one (8a)

Method A: LDA was prepared by adding BuLi (2.5 M in hexanes, 5.63 mL, 14.08 mmol) to diisopropylamine (1.57 g, 15.52 mmol) in THF (50 mL) cooled in an ice–salt bath, under nitrogen, and stirring for 10 min. This was then added to the isoxazolopyridone **6** (1.02 g, 6.70 mmol) and TMEDA (1.64 g, 14.11 mmol) in THF (120 mL) cooled in an ice–salt bath, under nitrogen, and the reaction mixture was stirred at this temperature for 20 min. Benzaldehyde (2.49 g, 23.46 mmol) was then added and the reaction mixture was stirred for 1 h at ice–salt bath temperature and then for 4 d at 20 °C. Hydrochloric acid (1 M, 100 mL) was added and the reaction mixture extracted with EtOAc (6 × 100 mL). The organic phase was washed with saturated aq $NaHCO_3$ (200 mL), saturated brine (200 mL), dried ($MgSO_4$) and concentrated under reduced pressure. Column chromatography (CH_2Cl_2/MeOH 100:1) yielded **8a** (0.85 g, 53%) as an off-white solid, which was recrystallised as colourless crystals; mp 212–214 ºC (CH_2Cl_2/MeOH); and recovered **6** (0.17 g, 17%) as an off-white solid.

^{1}H NMR (300 MHz, $CDCl_3$) δ 3.04 (t, *J* = 6.6 Hz, 2H, CH_2CH$_2$N), 3.64 (dt, *J* = 2.3, 6.6 Hz, 2H, CH$_2$CH_2N), 6.45 (br s, 1H, NH), 7.34–7.44 (m, 3H, Ar-H), 7.48 (d, *J* = 16.5 Hz, 1H, C*H*=CHPh), 7.60–7.66 (m, 2H, Ar-H), 7.79 (d, *J* = 16.5 Hz, 1H, CH=C*H*Ph); ^{13}C NMR (75 MHz, $CDCl_3$)δ 21.4 (*C*H$_2$CH$_2$N), 40.6 (CH$_2$*C*H$_2$N), 107.1 (C-3a), 112.1 (*C*H=CHPh), 127.9, 128.9, 130.0 (3 × Ar-CH), 135.18 (Ar-*C*), 139.2 (CH=*C*HPh), 161.0 (C-7a), 163.2 (C-3), 168.5 (CONH); IR (nujol): 3206, 3057, 1674, 1600, 1501, 1342, 1332, 1062, 972 cm^{-1}; EIMS *m*/*z* (%): 240 (100) [M]$^+$, 211 (16), 138 (23), 131 (50), 115 (25), 103 (92), 82 (30), 77 (92), 63 (17), 51 (39); HRMS (*m*/*z*): [M + H]$^+$ calcd for $C_{14}H_{12}N_2O_2$: 241.0971; found, 241.0977; Anal. calcd for $C_{14}H_{12}N_2O_2$: C, 69.99; H, 5.03; N, 11.66; found: C, 69.63; H, 4.72; N, 11.30.

Crystal data for **8a**: Data were collected on a Bruker Nonius KappaCCD with a Mo rotating anode generator; standard procedures were followed. $C_{14}H_{12}N_2O_2$, M = 240.26, colourless block, 0.40 × 0.30 × 0.30 mm; monoclinic, space group $P2_1/c$; a = 8.0372(3), b = 7.2024(3), c = 20.5918(4) Å, α = 90.00°, β = 100.438(3)°, γ = 90.00°, U = 1172.27(8) Å^3, T = 120(2) K, μ = 0.093 mm^{-1}, D_c = 1.361 Mg m^{-3}, Z = 4; 4292 reflections measured, 1638 unique (R_{int} = 0.0203), The final R_1 values were 0.0848 (I>2σ(I)). The final $wR(F_2)$ values were 0.2416 (I>2σ(I)). The final R_1 values were 0.0927 (all data). The final $wR(F_2)$ values were 0.2531 (all data). The goodness of fit on F_2 was 0.959.

3-[2-(4-Nitrophenyl)ethenyl]-4,5,6,7-tetrahydroisoxazolo[4,3-*c*]pyridin-4-one (8b)

Prepared according to *method A* for **8a**, by using BuLi (2.5 M in hexanes, 4.86 mL, 12.15 mmol), diisopropylamine (1.35 g, 13.34 mmol) in THF (45 mL), isoxazolopyridone **6** (0.88 g, 5.78 mmol) and TMEDA (1.41 g, 12.13 mmol) in THF (105 mL) and 4-nitrobenzaldehyde (3.06 g, 20.25 mmol). **8b** (0.86 g, 52%) was isolated as a yellow solid. Mp 278–280 °C; ^{1}H NMR (300 MHz, DMSO-d_6) δ 2.98 (t, J = 6.6 Hz, 2H, CH_2CH$_2$N), 3.47 (dt, J = 2.7, 6.6 Hz, 2H, CH$_2$CH_2N), 7.61 (d, J = 16.8 Hz, 1H, C*H*=CHPh), 7.97 (app d, J = 8.7 Hz, 2H, Ar-H), 7.99 (d, J = 16.8 Hz, 1H, CH=C*H*Ph), 8.08 (br s, 1H, NH), 8.28 (app d, J = 8.7 Hz, 2H, Ar-H); ^{13}C NMR (75 MHz, DMSO-d_6) δ 20.5 (*C*H$_2$CH$_2$N), 39.4 (CH$_2$*C*H$_2$N), 108.9 (C-3a), 115.6 (*C*H=CHPh), 124.1, 128.7 (2 × Ar-CH), 135.5 (CH=*C*HPh), 141.3, 147.5 (2 × Ar-C), 161.2 (C-7a), 161.8 (C-3), 165.8 (CONH); IR (nujol): 3196, 3080, 1667, 1645, 1606, 1595, 1514, 1340, 1265 cm^{-1}; EIMS m/z (%): 285 (47) [M]$^+$, 176 (27), 152 (23), 120 (25), 102 (37), 91 (58), 77 (45), 65 (29), 51 (36), 44 (100); HRMS (m/z): [M + H]$^+$ calcd for $C_{14}H_{11}N_3O_4$: 286.0822; found, 286.0827.

3-[2-(4-Bromophenyl)ethenyl]-4,5,6,7-tetrahydroisoxazolo[4,3-*c*]pyridin-4-one (8c) and 3-[2-hydroxy-2-(4-bromophenyl)ethyl]-4,5,6,7-tetrahydroisoxazolo[4,3-*c*]pyridin-4-one (7c)

Prepared according to *method A* for **8a**, by using BuLi (2.5 M in hexanes, 1.77 mL, 4.43 mmol), diisopropylamine (0.49 g, 4.84 mmol) in THF (20 mL), isoxazolo-pyridone **6** (0.32 g, 2.10 mmol) and TMEDA (0.51 g, 4.39 mmol) in THF (45 mL) and 4-bromobenzaldehyde (1.36 g, 7.35 mmol), with the reaction mixture stirred for 1 h at ice–salt bath temperature and then 2 d at 20 °C, to yield **8c** (0.10 g, 15%) as an off-white solid; mp 267 °C (decomp.) and **7c** (0.25 g, 35%) as a white solid; mp 189–191 °C.

For **8c**: ^{1}H NMR (300 MHz, $CDCl_3$) δ 3.05 (t, *J* = 6.5 Hz, 2H, CH_2CH$_2$N), 3.64 (dt, *J* = 2.6, 6.5 Hz, 2H, CH$_2$CH_2N), 6.02 (br s, 1H, NH), 7.45 (d, *J* = 16.6 Hz, 1H, C*H*=CHPh), 7.51 (m, 4H, Ar-H), 7.72 (d, *J* = 16.6 Hz, 1H, CH=C*H*Ph); ^{13}C NMR (75 MHz, $CDCl_3$) δ 21.4 (*C*H$_2$CH$_2$N), 40.7 (CH$_2$*C*H$_2$N), 107.3 (C-3a), 112.7 (*C*H=CHPh), 124.2, 129.3, 132.1 (3 × Ar-CH), 134.1 (Ar-C), 137.9 (CH=*C*HPh), 160.9 (C-7a), 162.9 (C-3), 168.2 (CONH); EIMS *m*/*z* (%): 320 (21), 318 (21) $[M]^+$, 149 (39), 102 (39), 44 (100); HRMS *m*/*z*: $[M]^+$ calcd for $C_{14}H_{11}{}^{79}BrN_2O_2$: 318.0004; found, 317.9999.

For **7c**: ^{1}H NMR (300 MHz, DMSO-d_6) δ 2.88 (t, *J* = 6.5 Hz, 2H, CH_2CH$_2$N), 3.30 (m, 2H, CH_2CHOH), 3.38 (m, 2H, CH$_2$CH_2N), 5.06 (m, 1H, CH$_2$C*H*OH), 5.76 (d, *J* = 5.0 Hz, 1H, CH$_2$CHO*H*), 7.29, 7.51 (each m, 2H, Ar-H), 7.84 (br s, 1H, NH); ^{13}C NMR (75 MHz, DMSO-d_6) δ 20.5 (*C*H$_2$CH$_2$N), 36.0 (*C*H$_2$CHOH), 39.5 (CH$_2$*C*H$_2$N), 69.7 (CH$_2$*C*HOH), 108.5 (C-3a), 120.0 (Ar-C), 127.8, 130.9 (2 × Ar-CH), 143.8 (Ar-C), 160.8 (C-7a), 161.6 (C-3), 171.2 (CONH); IR (nujol): 3590, 3390 br, 3221, 1666,

1614, 1514, 1342, 1088, 1077, 1058, 1011 cm^{-1}; CIMS *m*/*z* (NH_3, %): 339 (33), 337 (32) $[M + H]^+$, 259 (21), 155 (57), 77 (90), 58 (100); HRMS (*m*/*z*): $[M + H]^+$ calcd for $C_{14}H_{13}{}^{79}BrN_2O_3$: 337.0183; found, 337.0177.

3-[2-(2,4-Dichlorophenyl)ethenyl]-4,5,6,7-tetrahydroisoxazolo[4,3-*c*]pyridin-4-one (8d) and 3-[2-hydroxy-2-(2,4-dichlorophenyl)ethyl]-4,5,6,7-tetrahydroisoxazolo[4,3-*c*]pyridin-4-one (7d)

Prepared according to *method A* for **8a**, by using BuLi (2.5 M in hexanes, 4.64 mL, 11.60 mmol), diisopropylamine (1.29 g, 12.75 mmol) in THF (40 mL), isoxazolo-pyridone **6** (0.84 g, 5.52 mmol) and TMEDA (1.35 g, 11.62 mmol) in THF (100 mL) and 2,4-dichlorobenzaldehyde (3.38 g, 19.31 mmol) to yield **8d** (0.38 g, 22%) as an off-white solid; mp 222–223 °C; and **7d** (0.93 g, 51%) as a white solid; mp 178 °C.

For **8d**: ^{1}H NMR (300 MHz, DMSO-d_6)δ 2.97 (t, *J* = 6.6 Hz, 2H, CH_2CH$_2$N), 3.46 (dt, *J* = 2.7, 6.6 Hz, 2H, CH$_2$CH_2N), 7.49 (d, *J* = 16.6 Hz, 1H, C*H*=CHPh), 7.52 (m, 1H, Ar-H), 7.75 (d, *J* = 2.2 Hz, 1H, Ar-H), 7.96 (d, *J* = 8.6 Hz, 1H, Ar-H), 8.03 (br s, 1H, CONH), 8.21 (d, *J* = 16.6 Hz, 1H, CH=C*H*Ph); ^{13}C NMR (75 MHz, DMSO-d_6) δ 20.6 (*C*H$_2$CH$_2$N), 39.5 (CH$_2$*C*H$_2$N), 108.7 (C-3a), 115.4 (*C*H=CHPh), 128.1, 128.8, 129.5 (3 × Ar-CH), 131.8 (Ar-C), 132.3 (CH=*C*HPh), 134.2, 134.9 (2 × Ar-C), 161.3 (C-7a), 161.9 (C-3), 166.3 (CONH); IR (nujol): 3196, 3080, 1684, 1629, 1600, 1584, 1348, 1332, 1075, 1049, 984 cm^{-1}; EIMS *m*/*z* (%): 310 (10), 308 (15) $[M]^+$, 273 (42), 173 (35), 152 (100), 123 (41), 111 (56), 99 (39), 75 (60), 52 (32); HRMS (*m*/*z*): $[M + H]^+$ calcd for $C_{14}H_{10}{}^{35}Cl_2N_2O_2$: 309.0192; found, 309.0201.

For **7d**: ^{1}H NMR (300 MHz, MeOH-d_4) δ 2.95 (t, *J* = 6.5 Hz, 2H, CH_2CH$_2$N), 3.40–3.54 (m, 4H, CH_2CHOH & CH$_2$CH_2N), 5.53 (dd, *J* = 5.2, 7.4 Hz, 1H, CH$_2$C*H*OH), 7.33 (m, 1H, Ar-H), 7.40 (d, *J* = 1.8 Hz, 1H, Ar-H), 7.57 (d, *J* = 8.4 Hz, 1H, Ar-H); ^{13}C NMR

(75 MHz, MeOH-d_4) δ 21.9 (CH_2CH_2N), 35.6 (CH_2CHOH), 41.4 (CH_2CH_2N), 68.8 (CH_2CHOH), 110.3 (C-3a), 128.6, 129.9, 130.0 (3 × Ar-CH), 133.7, 134.9, 141.2 (3 × Ar-C), 162.5 (C-7a), 164.8 (C-3), 172.6 (CONH); IR (nujol): 3400 br, 3203, 1688, 1633, 1145, 1063, 1048 cm^{-1}; ESIMS *m*/*z* (%): 351 (23), 349 (34) $[M + Na]^+$, 329 (24), 327 (38), 151 (27), 119 (65), 105 (34), 82 (100), 55 (16); HRMS (*m*/*z*): $[M + H]^+$ calcd for $C_{14}H_{12}{}^{35}Cl_2N_2O_3$: 327.0298; found, 327.0308; Anal. calcd for $C_{14}H_{12}Cl_2N_2O_3$: C, 51.40; H, 3.70; N, 8.56; found: C, 51.29; H, 3.55; N, 8.49.

3-(3,3-Dimethyl-1-butenyl)-4,5,6,7-tetrahydroisoxazolo[4,3-*c*]pyridin-4-one (8e) and 3-(2-hydroxy-3,3-dimethylbutyl)-4,5,6,7-tetrahydroisoxazolo[4,3-*c*]pyridin-4-one (7e)

Prepared according to *method A* for **8a**, by using BuLi (2.5 M in hexanes, 4.75 mL, 11.88 mmol), diisopropylamine (1.32 g, 13.04 mmol) in THF (45 mL), isoxazolo-pyridone **6** (0.86 g, 5.65 mmol) and TMEDA (1.38 g, 11.88 mmol) in THF (100 mL) and 2,2-dimethylpropanal (1.70 g, 19.74 mmol). Column chromatography (CH_2Cl_2/MeOH 50:1) yielded **8e** (0.05 g, 4%) as an off-white solid; mp 192–193 °C; and **7e** (0.74 g, 55%) as an off-white solid; mp 172–173 °C.

For **8e**: ^1H NMR (300 MHz, $CDCl_3$) δ 1.16 (s, 9H, $C(CH_3)_3$), 3.00 (t, *J* = 6.5 Hz, 2H, CH_2CH_2N), 3.60 (dt, *J* = 2.8, 6.5 Hz, 2H, CH_2CH_2N), 6.72 (br s, 1H, NH), 6.75, 7.04 (each d, *J* = 16.5 Hz, 1H, CH=CH); ^{13}C NMR (75 MHz, $CDCl_3$) δ 21.4 (CH_2CH_2N), 28.8 ($C(CH_3)_3$), 34.4 ($C(CH_3)_3$), 40.5 (CH_2CH_2N), 106.1 (C-3a), 110.6 ($CH=CHC(CH_3)_3$), 154.0 ($CH=CHC(CH_3)_3$), 160.8 (C-7a), 163.4 (C-3), 169.0 (CONH); IR (nujol): 3220, 3090, 1682, 1665, 1603, 1341 cm^{-1}; EIMS *m*/*z* (%): 220 (30) $[M]^+$, 205 (21), 177 (26), 110 (38), 67 (45), 41 (100); HRMS (*m*/*z*): $[M + H]^+$ calcd for $C_{12}H_{16}N_2O_2$: 221.1284; found: 221.1286.

For **7e**: ^{1}H NMR (300 MHz, $CDCl_3$) δ 0.99 (s, 9H, C(CH_3)$_3$), 3.03 (m, 2H, CH_2CH_2N), 3.12, 3.24 (each m, 1H, CH_2CHOH), 3.61 (m, 3H, CH_2CH_2N and CH_2CHOH), 4.09 (d, J = 5.9 Hz, 1H, CH_2CHOH), 6.35 (br s, 1H, NH); ^{13}C NMR (75 MHz, $CDCl_3$) δ 21.3 (CH_2CH_2N), 25.5 (C(CH_3)$_3$), 30.3 (CH_2CHOH), 35.5 ($C(CH_3)_3$), 40.6 (CH_2CH_2N), 78.1 (CH_2CHOH), 108.8 (C-3a), 160.5 (C-7a), 164.1 (C-3), 174.6 (CONH); IR (nujol): 3452, 3225, 1672, 1631, 1517, 1338, 1133, 1081, 1059 cm^{-1}; EIMS *m*/*z* (%): 239 (4) [M + H]$^+$, 181 (93), 152 (100), 139 (28), 123 (40), 57 (76), 41 (99); HRMS (*m*/*z*): [M + H]$^+$ calcd for $C_{12}H_{18}N_2O_3$: 239.1390; found, 239.1389.

3-(4-Phenyl-1,3-butadienyl)-4,5,6,7-tetrahydroisoxazolo[4,3-*c*]pyridin-4-one (8f)

Prepared according to *method A* for **8a**, by using BuLi (2.5 M in hexanes, 5.24 mL, 13.10 mmol), diisopropylamine (1.46 g, 14.43 mmol) in THF (50 mL), the isoxazolo-pyridone **6** (0.95 g, 6.24 mmol) and TMEDA (1.52 g, 13.08 mmol) in THF (110 mL) and 3-phenylpropenal (2.89 g, 21.87 mmol). Column chromatography (EtOAc/hexane 8:2) yielded **8f** (0.20 g, 12%) as a yellow solid; mp 235–238 °C; and recovered **6** (0.13 g, 14%) as an off-white solid.

^{1}H NMR (300 MHz, $CDCl_3$) δ 3.03 (t, J = 6.5 Hz, 2H, CH_2CH_2N), 3.61 (dt, J = 2.7, 6.5 Hz, 2H, CH_2CH_2N), 5.99 (br s, 1H, NH), 6.88–7.06 (m, 3H, Ar-H and 2 × =CH), 7.27–7.63 (m, 6H, 4 × Ar-H and 2 × =CH); ^{13}C NMR (75 MHz, $CDCl_3$) δ 21.4 (CH_2CH_2N), 40.6 (CH_2CH_2N), 106.8 (C-3a), 115.3 (=CH), 127.1, 127.6, 128.8 (3 × Ar-CH), 128.9 (=CH), 136.3 (Ar-C), 139.4, 139.6 (2 × =CH), 160.8 (C-7a), 163.0 (C-3), 168.5 (CONH); IR (nujol): 3202, 3060, 1681, 1633, 1585, 1575, 1064, 1044 cm^{-1}; EIMS *m*/*z* (%): 266 (35) [M]$^+$, 138 (22), 128 (100), 115 (34), 102 (22), 82 (43), 77 (34), 63 (19), 51 (37), 44 (23); HRMS (*m*/*z*): [M + H]$^+$ calcd for $C_{16}H_{14}N_2O_2$: 267.1128; found, 267.1130.

3-(2-Phenylethenyl)-4,5,6,7-tetrahydroisoxazolo[4,3-*c*]pyridin-4-one (8a) via 3-(2-Hydroxy-2-phenylethyl)-4,5,6,7-tetrahydroisoxazolo[4,3-*c*]pyridin-4-one (7a)

Method B: To *n*-BuLi (2.5 M in hexanes; 0.6 mL, 1.5 mmol) in THF at −78 °C was added isoxazolopyridone **6** (100 mg, 0.65 mmol). The solution was stirred for 1 h at −78 °C followed by the addition of benzaldehyde (1.0 mL, 1.0 mmol). After being stirred for a further 10 min, the reaction was quenched with water (20 mL), and the mixture was acidified with hydrochloric acid (2 M; 20 mL) at −78 °C and extracted with EtOAc (3 × 30 mL). The combined organic layers were washed with brine (50 mL), dried ($MgSO_4$), filtered and the solvent was removed under reduced pressure. Column chromatography (EtOAc/PE 1:1) afforded **7a** (0.12 g, 70%) as a white solid; mp 174–175 °C.

^{1}H NMR (400 MHz, $CDCl_3$) δ 3.05 (t, *J* = 6.4 Hz, 2H, CH_2CH_2N), 3.50 (m, 2H, CH_2CHOH), 3.62 (td, *J* = 2.8, 6.4 Hz, 2H, CH_2CH_2N), 5.14–5.18 (m, 1H, CH_2CHOH), 6.05 (br, 1H, NH), 7.29 (t, *J* = 6.4 Hz, 1H, Ar-CH), 7.36 (t, *J* = 6.4 Hz, 2H, Ar-CH), 7.41 (d, *J* = 6.4 Hz, 2H, Ar-CH); ^{13}C NMR (100 MHz, $CDCl_3$) δ 21.2 (CH_2CH_2N), 37.3 (CH_2CHOH), 40.6 (CH_2CH_2N), 72.2 (CH_2CHOH), 110.0 (C-3a), 125.5, 127.8, 128.5 (3 × Ar-CH), 143.7 (Ar-C), 160.4 (C-7a), 163.8 (C-3), 172.5 (CONH); IR (CH_2Cl_2): 3475, 3215, 3050, 1672, 1620 1479, 1451, 1341, 1134, 1052 cm^{-1}; HRMS (*m*/*z*): [M]$^+$ calcd for $C_{14}H_{14}N_2O_3$: 258.1004; found, 258.1001.

To **7a** (50 mg, 0.19 mmol) in toluene (30 mL) was added PTSA (48 mg, 0.25 mmol) and the mixture was heated overnight under Dean–Stark conditions. The reaction mixture was cooled, water (20 mL) was added, and the mixture was extracted with EtOAc (3 × 30 mL). The combined organic layers were washed with brine (50 mL), dried ($MgSO_4$), filtered and the solvent was removed under reduced pressure to

leave **8a** as a white solid (29 mg, 63%); mp 217–219 °C; data identical to material prepared by *method A* above.

Dehydration of aldol hydroxyl adducts 7c–e:

3-[2-(4-Bromophenyl)ethenyl]-4,5,6,7-tetrahydroisoxazolo[4,3-*c*]pyridin-4-one (8c)

Method C: Alcohol **7c** (0.29 g, 0.860 mmol) and PTSA (0.25 g, 1.31 mmol) in toluene (150 mL) was heated at reflux under Dean–Stark conditions for 24 h, after which more PTSA (0.25 g, 1.31 mmol) was added and the mixture heated for another 24 h, by which time none of the alcohol was present by TLC. The reaction mixture was cooled, concentrated under reduced pressure and the residue dissolved in EtOAc (60 mL). The EtOAc solution was washed with saturated aq $NaHCO_3$ (60 mL), brine (60 mL), dried ($MgSO_4$) and concentrated under reduced pressure to yield a brown solid. Column chromatography (CH_2Cl_2/MeOH 100:1) yielded **8c** (0.11 g, 41%) as an off-white solid, mp 267 °C (decomp.); data identical to material prepared by *method A.*

3-[2-(2,4-Dichlorophenyl)ethenyl]-4,5,6,7-tetrahydroisoxazolo[4,3-*c*]pyridin-4-one (8d)

Prepared according to *method C* for **8c**, by using alcohol **7d** (0.78 g, 2.38 mmol), PTSA (0.68 g, 3.57 mmol), toluene (350 mL), and more PTSA (3.30 g, 17.35 mmol) over 4.5 d, to yield **8d** (0.28 g, 38%) as an off-white solid, mp 222–223 °C; data identical to material prepared by *method A*.

3-(3,3-Dimethyl-1-butenyl)-4,5,6,7-tetrahydroisoxazolo[4,3-*c*]pyridin-4-one (8e)

Prepared according to *method C* for **8c**, by using alcohol **7e** (0.42 g, 1.76 mmol), PTSA (0.41 g, 2.16 mmol), toluene (300 mL), and then more PTSA (2.62 g, 13.77 mmol) over 7 d, to yield **8e** (0.17 g, 44%) as an off-white solid, mp 192–193 °C; data identical to material prepared by *method A*.

2-(1-Hydroxyprop-2-enyl)-3,4-dihydro-2*H*-naphthalen-1-one (13)

To diisopropylamine (4.6 mL, 33 mmol) in THF (50 mL) at −78 °C was added *n*-BuLi (2.5 M in hexanes; 13.2 mL, 33.1 mmol). The solution was stirred for 1 h at this temperature before α-tetralone (3.9 mL, 30 mmol) was added dropwise and stirring continued for a further 1 h. Freshly distilled propenal (2.2 mL, 33 mmol) was added to the resulting solution dropwise over 15 min at −78 °C. After being stirred for a further 30 min, the reaction mixture was quenched with water (10 mL) at −78 °C and neutralised (5 M hydrochloric acid). Colour change was observed at this point, from yellow-green to a blue-green solution. The mixture was extracted with EtOAc (3 × 30 mL), and the combined organic layers were washed with hydrochloric acid (2 M; 30 mL), saturated aq $NaHCO_3$ (50 mL) and brine (50 mL), dried ($MgSO_4$), filtered and the solvent was removed under reduced pressure. Column chromatography (EtOAc/PE 1:7) afforded **13** as a colourless oil (obtained as a single compound) (5.2 g, 86%).

^{1}H NMR (400 MHz, $CDCl_3$) δ 2.18 (m, 2H, COCHCH_2), 2.58 (m, 1H, COC*H*), 3.00 (m, 2H, COCHCH_2CH_2), 4.27 (s, 1H, OH), 4.50 (m, 1H, C*H*OH), 5.23 (d, *J* = 10.4 Hz, 1H, CH=C*H*H), 5.34 (d, *J* = 17.2 Hz, 1H, CH=CH*H*), 5.90 (m, 1H, C*H*=CH_2), 7.25 (d, *J* = 7.6 Hz, 1H, Ar-CH), 7.31, 7.49 (each t, *J* = 7.6, 1H, Ar-CH), 8.03 (d, *J* = 7.6 Hz, 1H, Ar-CH); ^{13}C NMR (100 MHz, $CDCl_3$) δ 25.7, 28.8 (2 × CH_2), 52.4 (CH), 73.9 (CHOH),

117.3 (CH=CH_2), 126.7, 127.4, 128.7 (3 × Ar-CH), 132.4 (Ar-C), 133.8 (Ar-CH), 137.8 (*C*H=CH_2), 144.3 (Ar-C), 201.6 (CO); IR (CH_2Cl_2): 3447, 2930, 1673, 1598, 1454 cm^{-1}; HRMS–ESI (*m*/*z*): $[M + H]^+$ calcd for $C_{13}H_{14}O_2$: 203.1066; found, 203.1065.

2-(1-Hydroxyprop-2-enyl)-1,2,3,4-tetrahydronaphthalen-1-ol (14)

To a stirred suspension of $LiAlH_4$ (0.61 g, 18 mmol) in THF (40 mL) was added hydroxyketone **13** (2.0 g, 9.9 mmol) in THF (5 mL) over 5 min at 0 °C and the mixture stirred for 30 min before being quenched with THF/water (1:1; 20 mL). Aq sodium hydroxide (15% w/v; 20 mL) was added and the mixture was extracted with EtOAc (3 × 30 mL). The organic layers were combined, dried ($MgSO_4$), filtered and the solvent was removed under reduced pressure. Column chromatography (EtOAc/PE 1:4) gave **14** (1.5 g, 75%) as a yellow oil.

For major isomer: ^{1}H NMR (400 MHz, $CDCl_3$) δ 1.71 (m, 2H, CHC*H*$_2$CH$_2$), 1.85 (m, 1H, C*H*CH$_2$CH$_2$), 2.75 (m, 2H, CHCH$_2$C*H*$_2$), 4.01, 4.78 (each m, 1H, C*H*OH), 5.24 (m, 2H, CH=C*H*$_2$), 5.86 (m, 1H, C*H*=CH$_2$), 7.01–7.53 (m, 4H, Ar-CH); ^{13}C NMR (100 MHz, $CDCl_3$) δ 23.6, 28.6 (2 × CH_2), 46.7 (CH), 68.5, 70.9 (2 × CHOH), 116.2 (CH=*C*H$_2$), 126.9, 127.2, 129.2, 129.8 (4 × Ar-CH), 136.4 (Ar-C), 137.9 (*C*H=CH$_2$), 138.6 (Ar-C); IR (CH_2Cl_2): 3352, 2925, 1490, 1431 cm^{-1}; HRMS–ESI (*m*/*z*): $[M + Na]^+$ calcd for $C_{13}H_{16}O_2$: 227.1043; found, 227.1042.

4-Ethenyl-4a,5,6,10b-tetrahydro-4*H*-naphtho[1,2-*d*][1,3]dioxin-2-one (15)

To 1,3-diol **14** (0.54 g, 2.7 mmol) and Et_3N (3.10 mL, 22.0 mmol) in dry CH_2Cl_2 (20 mL) at 0 °C was added dropwise methyl chloroformate (1.35 mL, 17.5 mmol). The mixture was stirred for 24 h at 23 °C and then the solvent concentrated under

reduced pressure. To the resulting mixture, hydrochloric acid (2 M; 50 mL) was added and the mixture was extracted with EtOAc (3 × 30 ml), the organic layers were combined, washed with brine (50 mL), dried ($MgSO_4$), filtered and the solvent was removed under reduced pressure. Column chromatography (EtOAc/PE 1:5) yielded **15** as a white solid (0.44 g, 74%), as a diastereoisomeric mixture; m.p. 150–152 °C. For major isomer: ^{1}H NMR (400 MHz, $CDCl_3$) δ 2.06 (m, 2H, CHCH_2CH$_2$), 2.95 (m, 3H, C*H*CH$_2$CH_2), 4.71 (m, 1H, C*H*CH=CH$_2$), 5.31 (d, *J* = 10.0 Hz, 1H, CHO), 5.48 (m, 2H, CH=CH_2), 5.93 (m, 1H, C*H*=CH$_2$), 7.14–7.61 (m, 4H, Ar-CH); IR (CH_2Cl_2): 2879, 1684, 1660, 1532, 1490, 1362 cm^{-1}; HRMS–ESI (*m*/*z*): $[M + NH_4]^+$ calcd for $C_{14}H_{14}O_3$: 248.1287; found, 248.1283.

2-(Hexa-3,5-dienyl)benzaldehyde (12)

To cyclic carbonate **15** (2.40 g, 10.4 mmol) in dry MeCN (20 mL) was added $[Pd_2(dba)_3]\cdot CHCl_3$ (0.54 g, 0.52 mmol) and the mixture stirred for 48 h at 23 °C. The reaction mixture was then diluted with Et_2O (10 mL) and filtered through a Celite pad. The filtrate was washed with saturated aq $NaHCO_3$ (30 mL), dried ($MgSO_4$), filtered and the solvent removed under reduced pressure. Column chromatography (EtOAc/PE 1:10) gave **12** (1.24 g, 65%) as a yellow oil.

^{1}H NMR (400 MHz, $CDCl_3$) δ 2.42 (m, 2H, ArCH$_2$CH_2), 3.14 (t, *J* = 7.6 Hz, 2H, ArCH_2CH$_2$), 4.99 (d, *J* = 10.0 Hz, 1H, CH=C*H*H), 5.12 (d, *J* = 16.8 Hz, 1H, CH=CH*H*), 5.75, 6.01 (each m, 1H, CH=CH), 6.30 (m, 1H, C*H*=CH$_2$), 7.26 (d, *J* = 7.6 Hz, 1H, Ar-CH), 7.38, 7.49 (each t, *J* = 7.6 Hz, 1H, Ar-CH), 7.84 (d, *J* = 7.6 Hz, 1H, Ar-CH), 10.25 (s, 1H, CHO); ^{13}C NMR (100 MHz, $CDCl_3$) δ 32.4, 32.8 (2 × CH$_2$), 115.6 (CH=CH_2), 126.7, 131.1, 131.9, 132.2 (4 × Ar-CH), 133.5 (CH), 133.7 (Ar-C), 133.8, 136.9 (2 × CH), 144.4 (Ar-C), 192.4 (CHO); IR (CH_2Cl_2): 3442, 2358, 1696, 1598,

1451 cm^{-1}; HRMS–ESI (*m*/*z*): $[M + NH_4]^+$ calcd for $C_{13}H_{14}O$: 204.1388; found, 204.1380.

3-[2-(2-Hexa-3,5-dienylphenyl)-2-hydroxyethyl]-4,5,6,7-tetrahydroisoxazolo[4,3-*c*]pyridin-4-one (16)

Prepared according to *method B* for **7a**, by using isoxazolopyridone **6** (50 mg, 0.33 mmol), *n*-BuLi (2.5 M in hexanes; 0.66 mL, 1.6 mmol) and aldehyde **12** (0.61 g, 3.3 mmol) to yield **16** as a white solid (58 mg, 53%); mp 123–125 °C.

^1H NMR (400 MHz, $CDCl_3$) δ 2.49 (m, 2H, $ArCH_2CH_2$), 2.89 (t, *J* = 7.6 Hz, 2H, $ArCH_2CH_2$), 3.01 (t, *J* = 6.4 Hz, 2H, CH_2CH_2N), 3.38–3.51 (m, 2H, $CH(OH)CH_2$), 3.62 (t, *J* 6.4, 2H, CH_2CH_2N), 4.58 (br, 1H, CHO*H*), 4.99 (d, *J* = 10.0 Hz, 1H, CH=C*H*H), 5.12 (d, *J* = 16.8 Hz, 1H, CH=CH*H*), 5.39 (m, 1H, C*H*(OH)CH_2), 5.78, 6.08 (each m, 1H, CH=CH), 6.35 (m, 1H, C*H*=CH_2), 7.18–7.29 (m, 3H, Ar-CH), 7.56 (m, 1H, Ar-CH); ^{13}C NMR (100 MHz, $CDCl_3$) δ 21.3, 31.8, 34.3, 36.7, 40.6 (5 × CH_2), 68.5 (CHOH), 109.1 (C-3a), 115.5 (CH=CH_2), 125.5, 126.7, 127.8, 129.5 (4 × Ar-CH), 131.7, 133.9, 137.0 (3 × CH), 137.9, 140.9 (2 × Ar-C), 160.5 (C-7a), 163.8 (C-3), 172.7 (CONH); IR (CH_2Cl_2): 3314, 2951, 1666, 1478, 1334 cm^{-1}; HRMS–ESI (*m*/*z*): $[M + H]^+$ calcd for $C_{20}H_{22}N_2O_3$: 339.1703; found, 339.1707.

3-[2-(2-Hexa-3,5-dienylphenyl)ethenyl]-4,5,6,7-tetrahydroisoxazolo[4,3-*c*]pyridin-4-one (11)

To hydroxy adduct **16** (50 mg, 0.15 mmol) in toluene (5 mL) was added PTSA (42 mg, 0.22 mmol), and the mixture was heated for 4 h under Dean–Stark conditions. The mixture was cooled and water (10 mL) added, it was then extracted with EtOAc (4 × 30 mL), and the combined organic layers were washed with brine

(50 mL), dried ($MgSO_4$) and filtered, and the solvent was removed under reduced pressure. Column chromatography (EtOAc/PE 1:1) yielded **11** as a white solid (37 mg, 78%); mp 160–164 °C.

^{1}H NMR (400 MHz, $CDCl_3$) δ 2.49 (q, *J* = 7.2 Hz, 2H, $ArCH_2CH_2$), 2.90 (t, *J* = 7.2 Hz, 2H, $ArCH_2CH_2$), 3.04 (t, *J* = 6.8 Hz, 2H, CH_2CH_2N), 3.64 (t, *J* = 6.8 Hz, 2H, CH_2CH_2N), 4.98 (d, *J* = 10.0 Hz, 1H, CH=C*H*H), 5.12 (d, *J* = 16.8 Hz, 1H, CH=CH*H*), 5.76, 6.08 (each m, 1H, CH=CH), 6.29 (m, 1H, C*H*=CH_2), 7.17–7.32 (m, 3H, Ar-CH), 7.38 (d, *J* = 16.4 Hz, 1H, ArCH=C*H*), 7.74 (m, 1H, Ar-CH), 8.19 (d, *J* = 16.4 Hz, 1H, ArC*H*=CH); ^{13}C NMR (100 MHz, $CDCl_3$) δ 21.4, 33.2, 34.4, 40.6 (4 × CH_2), 106.9 (C-3a), 113.2 (CH), 115.4 (CH=CH_2), 126.3, 126.7, 129.9, 130.1 (4 × Ar-CH), 131.9, 133.7, 137.0, 137.04 (4 × CH), 141.3, 161.0 (2 × Ar-C), 163.3 (C-7a), 168.9 (C-3), 175.5 (CONH); IR (CH_2Cl_2): 3019, 2956, 2385, 1671, 1597, 1482, 1336 cm^{-1}; HRMS–ESI (*m*/*z*): $[M + H]^+$ calcd for $C_{20}H_{20}N_2O_2$: 321.1597; found, 321.1607.
